# A20 and TNIP-3 Reduce NF-κB-Mediated Paracrine Responses to Hypoxia/Hyperglycemia-Induced Endothelial Senescence

**DOI:** 10.3390/cells14231908

**Published:** 2025-12-02

**Authors:** Lara Russo, Serena Babboni, Serena Del Turco, Giuseppina Basta

**Affiliations:** Institute of Clinical Physiology, CNR San Cataldo Research Area, Via Moruzzi 1, 56124 Pisa, Italy; lararusso@cnr.it (L.R.); serenababboni@cnr.it (S.B.); serena@ifc.cnr.it (S.D.T.)

**Keywords:** endothelial cells, TNIP-3, A20, SASP, NF-κB, aging, hypoxia, hyperglycemia

## Abstract

**Highlights:**

**What are the main findings?**

- Hypoxia, alone or combined with hyperglycemia, induces endothelial cell senescence without activating the classical pro-inflammatory SASP.
- This condition is associated with the upregulation of A20 and TNIP-3, suggesting a deviation from canonical senescence programs.

**What are the implications of the main findings?**

- The non-canonical senescence profile observed under hypoxia indicates that endothelial senescence may be more heterogeneous than previously recognised.
- The functional significance of A20 and TNIP-3 upregulation in this context remains to be clarified and represents an important direction for future studies.

**Abstract:**

Background: Hypoxia and ageing both involve impaired oxygen delivery, leading to oxidative damage, and endothelial cell (EC) dysfunction. In the presence of chronic hyperglycemia, these effects are amplified, accelerating EC senescence and vascular impairment. Methods: We assessed key mediators of inflammatory signalling and senescence, as well as transcriptional regulators responsive to oxidative stress in ECs exposed to high glucose (30.5 mmol/L) for 72 h under either normoxia (21% O_2_) or prolonged (16 h) hypoxia (2% O_2_) followed by 2 h of reoxygenation. Results: ECs exposed to high glucose and hypoxia developed a senescent phenotype, as indicated by increased expression of p21 and p16, and elevated β-galactosidase staining. Interestingly, hypoxia-induced senescence did not coincide with the classical senescence-associated secretory phenotype (SASP). Compared to normoxia, ECs exposed to hypoxia, particularly under high-glucose conditions, showed reduced NF-κB-driven proinflammatory secretome (MCP-1, IL-6, IL-8), downregulation of the NF-κB p50 subunit, and simultaneous upregulation of the angiogenic factor VEGF-A with downregulation of YAP-1, a key regulator of cell survival. Notably, we observed a strong upregulation of A20 and TNIP-3, two well-characterized negative regulators of NF-κB signalling. Conclusions: Hypoxia-induced senescence did not trigger a typical inflammatory SASP. Although ECs enter a senescent state, they activate an anti-inflammatory response, suppressing NF-κB signalling and increasing the expression of its inhibitors, A20 and TNIP-3. This may reflect a non-canonical senescence response whose functional significance remains to be determined.

## 1. Introduction

Endothelial cell (EC) senescence is increasingly recognised as a key contributor to the onset and progression of age-related vascular and neurodegenerative diseases, including atherosclerosis, vascular dementia, and Alzheimer’s disease [1,2,3,4,5]. Senescent ECs acquire a proinflammatory, pro-thrombotic, and pro-oxidative phenotype collectively referred to as the senescence-associated secretory phenotype (SASP) [6,7]. This shift compromises vascular homeostasis by altering nitric oxide bioavailability, promoting leukocyte adhesion, and impairing angiogenic capacity. As a result, endothelial senescence contributes to blood–brain barrier dysfunction, vascular stiffening, and plaque formation, all of which are hallmarks of vascular aging and chronic inflammation [1,8,9,10].

Multiple stressors can induce EC senescence, among which hypoxia and hyperglycemia are particularly relevant in human pathology [11,12,13]. Chronic hyperglycemia, a key feature of diabetes mellitus, disturbs mitochondrial metabolism and increases the production of reactive oxygen species (ROS) [7,14]. The resulting oxidative stress activates pro-inflammatory signalling cascades such as NF-κB and p38 MAPK, leading to upregulation of cell cycle inhibitors (p21 and p16) and an increased proportion of senescence-associated β-galactosidase (SA-β-gal)-positive cells [6,14,15]. Similarly, hypoxia, a condition of insufficient oxygen availability, impairs mitochondrial respiration and ATP generation [1,15,16]. It alters calcium homeostasis and stabilises hypoxia-inducible factors (HIF), which in turn affect gene expression related to inflammation, angiogenesis, and metabolism [15,16]. Prolonged hypoxia promotes ROS accumulation and activates stress-response pathways that accelerate cellular senescence [17,18].

Importantly, the coexistence of hypoxic and hyperglycemic conditions—common in ischemic diabetic tissues—can exacerbate endothelial injury and senescence [11]. This combination closely mirrors the multifactorial microenvironment observed in aging, metabolic disorders, and chronic vascular diseases [1,4,18]. Understanding how these stimuli modulate endothelial aging processes at molecular and functional levels is imperative for elucidating mechanisms of vascular degeneration and identifying potential therapeutic targets.

In this study, we investigated the combined effects of prolonged hypoxia and high-glucose exposure on the senescence of ECs. Specifically, we analysed the expression of key senescence markers (p21, p16, and SA-β-gal) together with the secretory profiles of pro-inflammatory mediators. Special attention was given to the regulation of the Nuclear Factor-κB (NF-κB) signalling pathway and its endogenous inhibitors, including A20 (Tumor Necrosis Factor Alpha-Induced Protein 3, *TNFAIP3*) and *TNFAIP3* Interacting Protein 3 (TNIP-3), which play pivotal roles in maintaining endothelial integrity and controlling inflammatory responses.

## 2. Materials and Methods

### 2.1. Cell Culture

Human umbilical vein ECs were obtained from PromoCell (Heidelberg, Germany). After thawing, cells were cultured in EC growth medium (PromoCell) supplemented with the manufacturer’s recommended growth factors and 1% penicillin–streptomycin. Cell culture flasks were pre-coated with 0.1% porcine gelatin (Sigma-Aldrich, St. Louis, MO, USA) for 30 min at 37 °C. After seeding, the cell medium was replaced after 24 h and subsequently renewed every 48 h. Cells were maintained at 37 °C in a humidified incubator under normoxic conditions (21% O_2_, 5% CO_2_). When cell monolayers reached approximately 80% confluence, they were detached using 0.25% trypsin–EDTA and reseeded as required. Cells at passage IV were used for all experimental treatments, and each culture plate, according to its geometry, was designated for specific downstream assays.

### 2.2. Experimental Design for Glucose and Hypoxia Treatments

Twenty-four hours after seeding, the expansion medium was replaced with a minimal growth medium (expansion medium supplemented with 2% fetal bovine serum and no growth factors). Cells were then treated for 72 h to either high glucose (30.5 mmol/L, hyperglycemia) or physiological glucose (5.5 mmol/L, normoglycemia).

To induce hypoxic conditions, a hypoxia incubator chamber (Stemcell Technologies, Vancouver, BC, Canada) was used during the final 24 h of treatment. After 48 h of exposure to the respective glucose conditions, cells were placed in the chamber, and a gas mixture of 2% O_2_, 5% CO_2_, and 93% N_2_ (Air Liquide, Milan, Italy) was flushed at a rate of 20 L/min for 7 min to establish a hypoxic environment. The chamber was then sealed and incubated overnight at 37 °C. Simultaneously, as a control, some cell plates were placed in an incubator at normoxic conditions (21% O_2_, 5% CO_2_, and 74% N_2_). The following day, hypoxic cell monolayers were reoxygenated (21% O_2_, 5% CO_2_, and 74% N_2_) for 2 h, and finally processed for RNA extraction. Cell culture supernatants were collected, centrifuged, and snap-frozen for later analysis. Unless otherwise specified, all experiments were performed under these exposure conditions.

### 2.3. Cell Viability Assays

An osmotic control (5.5 mmol/L glucose + 25 mmol/L mannitol) was tested only in preliminary experiments to assess potential osmotic toxicity. ECs were incubated for 72 h under the following conditions: 5.5 mmol/L glucose (physiological glycemia and osmolarity), 30.5 mmol/L glucose (hyperglycemia and high osmolarity), and 5.5 mmol/L glucose supplemented with 25 mmol/L mannitol (osmotic control). Cell viability was assessed by the MTT assay (Sigma-Aldrich). Briefly, after treatment, 10 µL of MTT solution (5 mg/mL in PBS) was added to each well of a 96-well plate and incubated for 4 h at 37 °C in 5% CO_2_. Formazan crystals were solubilised with 100 µL of 10% SDS in 0.01 M HCl, and absorbance was measured at 565 nm using a microplate reader (Tecan Infinite^®^ 200 PRO, Tecan Group Ltd., Männedorf, Switzerland).

Cell viability under normoxic and hypoxic conditions (with or without glucose) was determined by measuring the uptake of the membrane-impermeable dye Trypan Blue (Thermo Fisher Scientific, Waltham, MA, USA). Before detachment, the culture medium was collected to recover non-adherent cells, which were centrifuged and combined with the adherent fraction to ensure accurate total cell counts. Adherent cells cultured in six-well plates were then detached using 0.25% trypsin, resuspended in complete medium, and mixed 1:4 with Trypan Blue. Viable cells that excluded the dye were counted using a Neubauer hemocytometer. The number of viable cells per milliliter was calculated using the formula: viable cells/mL = (mean count per quadrant × 10^4^ × dilution factor). Counts were performed in technical triplicate for each experimental condition.

### 2.4. Senescence-Associated β-Galactosidase Activity by Flow Cytometry

SA-β-Gal activity was quantified using the CellEvent™ Senescence Green Flow Cytometry Assay Kit (Thermo Fisher Scientific), following the manufacturer’s instructions. This fluorogenic method allows for a more sensitive and accurate quantitative detection of SA-β-Gal activity at pH 6.0, compared to the traditional histochemical assay [19,20]. Briefly, cells were seeded in 12-well plates and treated according to the experimental design. After treatment, they were harvested, washed twice with PBS, and fixed in 2% paraformaldehyde for 10 min at room temperature. Cells were then washed with 1% BSA/PBS and incubated with the CellEvent™ Senescence Green probe (1:1000 dilution) for 2 h at 37 °C in a CO_2_-free incubator. After incubation, samples were washed, resuspended in PBS, and analysed immediately by flow cytometry (excitation/emission: 490/514 nm). A minimum of 5000 events per sample was recorded, and mean fluorescence intensity was used as a quantitative measure of SA-β-Gal activity. Unstained controls were included to define background fluorescence.

### 2.5. Multiplex Cytokine Quantification

Proinflammatory secretome in EC culture supernatants was quantified using the MILLIPLEX^®^ Human Cytokine/Chemokine/Growth Factor Panel A (Merck KGaA, Darmstadt, Germany), following the manufacturer’s instructions. This multiplex bead-based immunoassay is based on Luminex^®^ xMAP^®^ technology (Luminex Corporation, Austin, TX, USA), which enables the simultaneous quantification of multiple analytes in a single sample [21,22].

Briefly, cell culture supernatants were collected after experimental treatments, centrifuged at 1000× *g* for 10 min at 4 °C to remove debris, and stored at −80 °C until analysis. Standards and samples were incubated with fluorescent-coded magnetic beads pre-coated with target-specific capture antibodies. After washing, biotinylated detection antibodies and streptavidin–phycoerythrin (PE) conjugate were added sequentially to allow sandwich complex formation. The plates were read on a MAGPIX^®^ analyser (Luminex Corporation), which identifies each bead by its spectral signature and quantifies PE fluorescence intensity proportional to analyte concentration.

Data acquisition and analysis were performed using Belysa^®^ Immunoassay Curve Fitting Software (v1.2 for Windows, Cat. No. 40–122, Merck KGaA, Darmstadt, Germany). A five-parameter logistic (5-PL) regression model was applied to generate standard curves and interpolate sample concentrations, ensuring high accuracy and reproducibility across the dynamic range of detection.

### 2.6. Quantitative Reverse Transcription Polymerase Chain Reaction

Total RNA was extracted from ECs using the miRNeasy Mini Kit (Qiagen, Milan, Italy) according to the manufacturer’s protocol [22]. RNA concentration and purity were assessed using a NanoDrop spectrophotometer (Thermo Fisher Scientific), and samples with A260/280 ratios between 1.8–2.1 were considered suitable for cDNA synthesis.

Complementary DNA (cDNA) was synthesised from 1 μg of total RNA using the iScript cDNA Synthesis Kit (Bio-Rad, Hercules, CA, USA) following standard thermal cycling conditions. For the assessment of p16, p21, and A20, quantitative reverse transcription-polymerase chain reactions (qRT-PCR) were performed using SsoAdvanced™ Universal SYBR^®^ Green Supermix (Bio-Rad) in 20 μL reactions containing 10 ng of cDNA and 500 nM primers. β-actin was used as an endogenous reference to normalise gene expression. Primer sequences and annealing temperatures were as follows: β-actin (F: CACCATTGGCAATGAGCGGTTC; R: AGGTCTTTGCGGATGTCCACGT; 60 °C), p16 (F: GAGCAGCATGGAGCCTTC; R: CATCATCATGACCTGGATCG; 56 °C), p21 (F: GTCACTGTCTTGTACCCTTGTG; R: CGGCGTTTGGAGTGGTAGAAA; 62 °C), and A20 (F: AGAGAGATCACACCCCCAGC; R: TGCTCTCCAACACCTCTCCG; 61 °C).

For VEGF-A (Hs00900055_m1), *NFκB1* (Hs00765730_m1), TNIP-3 (Hs00375573_m1), β-actin (Hs01860665_g1), and YAP-1 (Hs00902712_g1), pre-optimised TaqMan Gene Expression Assays with TaqMan Fast Advanced Master Mix (Thermo Fisher Scientific) were used according to the manufacturer’s protocol. qRT-PCR (final volume: 20 µL) was performed in a Rotor-Gene Q thermocycler (QIAGEN, Hilden, Germany), and relative gene expression was calculated using the 2^−ΔΔCt^ method [23], ensuring normalisation to β-actin and comparison across experimental conditions.

### 2.7. Statistical Analysis

Experimental results were repeated thrice and reported as mean ± standard deviation. Data were analysed using two-way ANOVA followed by a Bonferroni post hoc test for multiple comparisons. The data were analysed using SPSS 26 (SPSS, Chicago, IL, USA). A *p*-value < 0.05 was considered significant.

## 3. Results

### 3.1. Preliminary Assessment of EC Viability Under Hyperglycemia, Osmolarity and Hypoxia

As a preliminary assessment, ECs were incubated for 72 h with 5.5 mmol/L glucose (normoglycemia), 30.5 mmol/L glucose (hyperglycemia), or 5.5 mmol/L glucose plus 25 mmol/L mannitol (osmotic control) to distinguish glucose-specific effects from those attributable to increased osmolarity. MTT assay-based cell viability was not significantly (*p* > 0.05 vs. normoglycemia) affected by any of the conditions tested (5.5 mmol/L glucose: 98.7 ± 1.0%; 30.5 mmol/L glucose: 97.4 ± 1.3%; 5.5 mmol/L glucose + 25 mmol/L mannitol: 97.6 ± 1.6%). Since osmotic treatment did not induce cytotoxicity, osmotic control was not included in subsequent experiments.

Furthermore, a Trypan Blue-based assessment of cell viability in ECs under normoxic, hyperglycemic, hypoxic, and combined hypoxic and hyperglycemic conditions yielded comparable results across all experimental settings. In normoxia, hyperglycemia, hypoxia and hypoxia + hyperglycemia, the total number of viable cells per well was (1.32 ± 0.2) × 10^6^, (1.3 ± 0.14) × 10^6^, (1.26 ± 0.15) × 10^6^, and (1.2 ± 0.25) × 10^6^, respectively. No significant differences were observed in total cell counts or viability percentage, indicating that neither hypoxia alone nor combined exposure (hypoxia + hyperglycemia) compromised EC survival.

### 3.2. Hypoxia and Hyperglycemia Increased Senescence-Associated β-Galactosidase Activity in ECs

**Figure 1:**
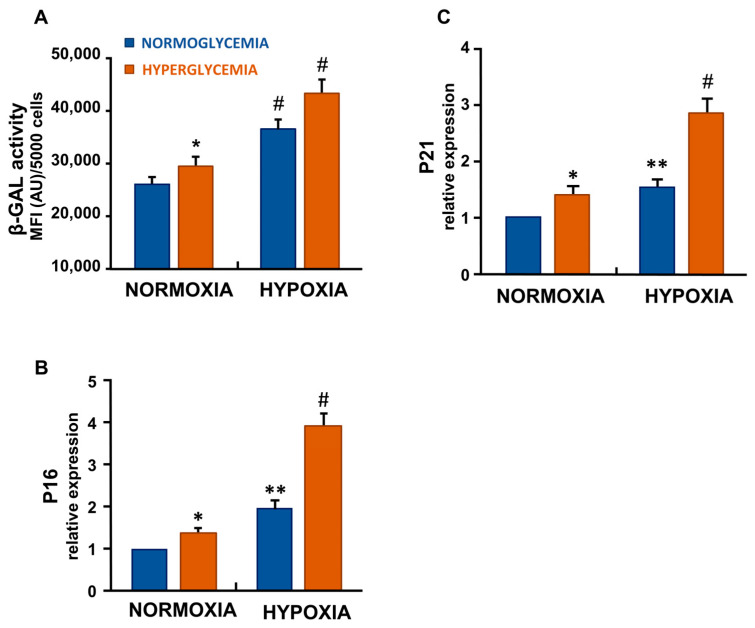
Hyperglycemia and hypoxia induced a senescence phenotype in ECs. (**A**) SA-β-gal activity increased under hyperglycemia and hypoxia compared to control. Dual treatment led to the maximum SA-β-gal signal. (**B**,**C**) Similarly, p16 and p21 mRNA expression increased, especially in the combined condition. Data represent mean ± SD of three independent experiments, each performed in duplicate [* *p* < 0.05, ** *p* < 0.01 and # *p* < 0.001 compared with control (normoxia with 5.5 mM glucose)].

SA-β-Gal activity, a marker of cellular senescence, was significantly elevated by hyperglycemia (*p* < 0.05), hypoxia (*p* < 0.001), and combined exposure (hypoxia + hyperglycemia) (*p* < 0.001) compared with normoxic, normoglycemic control (Figure 1A).

### 3.3. Hypoxia and High Glucose Increased the Gene Expression of p16 and p21 in ECs

Senescence was further evaluated by measuring the cyclin-dependent kinase inhibitors p16 and p21. Transcriptional analysis revealed an upregulation of both genes across all experimental conditions compared with normoxic, normoglycemic control (Figure 1B,C). p16 increased modestly under hyperglycemia (*p* < 0.05), but increased under hypoxia (*p* < 0.01), and under combined hyperglycemia and hypoxia (*p* < 0.001), compared with normoxic, normoglycemic control (Figure 1B). Similarly, p21 was slightly elevated under hyperglycemia (*p* < 0.05) but increased under hypoxia (*p* < 0.01) and under the combined condition (*p* < 0.001), compared with normoxic, normoglycemic control (Figure 1C).

### 3.4. Hypoxia Alone and Hypoxia Combined with High Glucose Did Not Elicit a Canonical Pro-Inflammatory SASP in ECs

**Figure 2:**
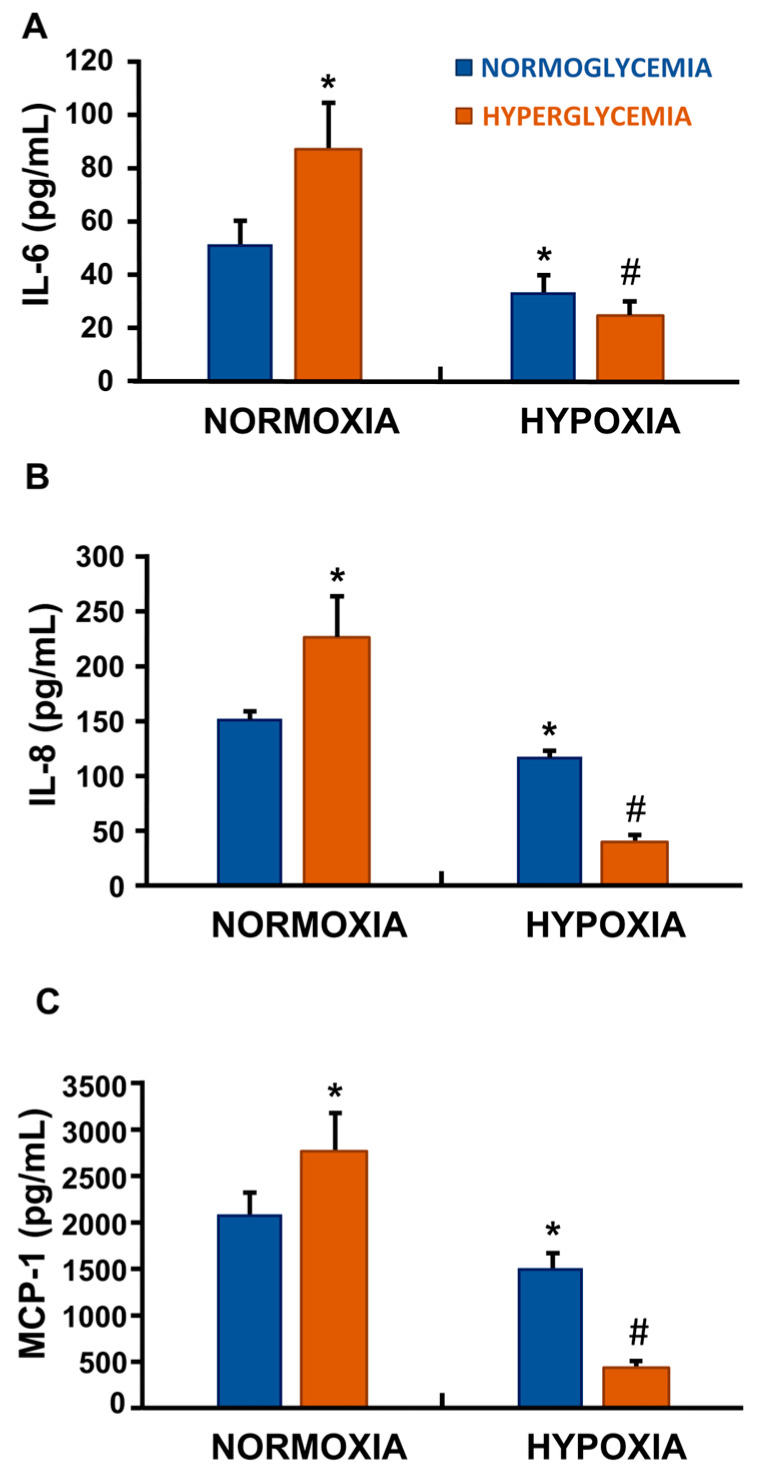
EC SASP during hyperglycemia and hypoxia. High glucose conditions alone promoted a senescent phenotype, as indicated by increased markers IL-6, IL-8, and MCP-1 in the supernatant (**A**–**C**). Conversely, exposure to hypoxia, alone or in combination with high glucose levels, significantly reduced their release into the supernatant (**A**–**C**). Data represent mean ± SD of three independent experiments, each performed in duplicate [* *p* < 0.05, and # *p* < 0.001 compared with control (normoxia with 5.5 mM glucose)].

Pro-inflammatory SASP mediates the paracrine effects of senescent cells on their surrounding tissue microenvironment. In our model, elevated glucose levels alone were sufficient to induce a clear senescent phenotype in ECs (*p* < 0.05 vs. normoxic, normoglycemic control) (Figure 2A–C). Unexpectedly, however, hypoxia—either alone or in combination with high glucose—attenuated the pro-inflammatory secretory phenotype, resulting in a marked reduction in the cytokines IL-6 and IL-8, as well as of the chemokine MCP-1 (Figure 2A–C).

### 3.5. Differential Regulation of VEGF-A and Yes-Associated Protein-1 (YAP-1) in Response to Hypoxia and High Glucose

**Figure 3:**
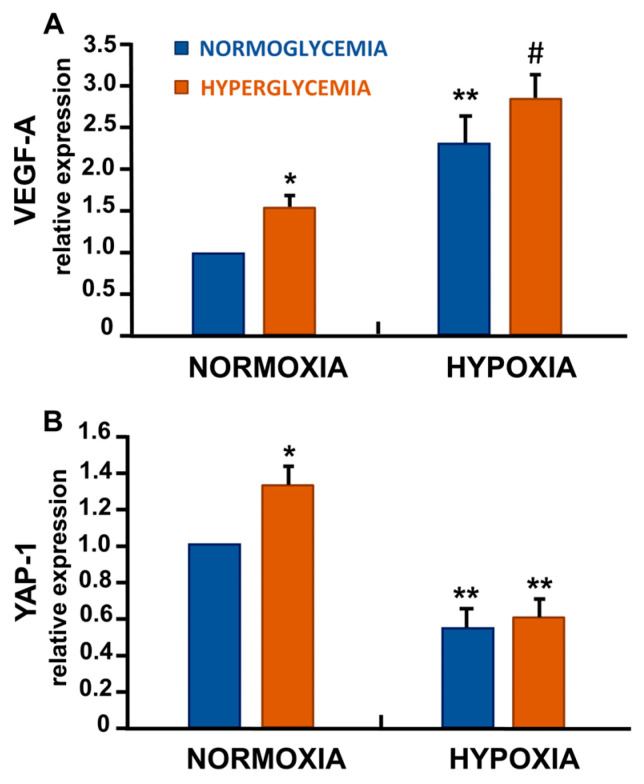
High glucose and hypoxia modulated VEGF-A and YAP-1 expressions. Exposure of ECs to high glucose and hypoxia, either alone or in combination, increased VEGF-A and decreased YAP-1 transcriptional expressions (**A**,**B**). Data represent mean ± SD of three independent experiments, each performed in duplicate [* *p* < 0.05, ** *p* < 0.01 and # *p* < 0.001 compared with control (normoxia with 5.5 mM glucose)].

As expected, high glucose and hypoxia, either alone (*p* < 0.05 and *p* < 0.01, respectively) or in combination (*p* < 0.001), significantly induced transcriptional upregulation of the angiogenic factor VEGF-A (Figure 3A). Furthermore, we analysed the YAP-1 levels, a key regulator of the Hippo signalling pathway, which is involved in cell growth and survival. Notably, YAP-1 mRNA expression was significantly reduced under hypoxia-induced senescence (*p* < 0.01) (Figure 3B).

### 3.6. Hypoxia and High Glucose Shifted NF-κB Signalling by Decreasing p50 and Increasing A20/TNIP-3

**Figure 4:**
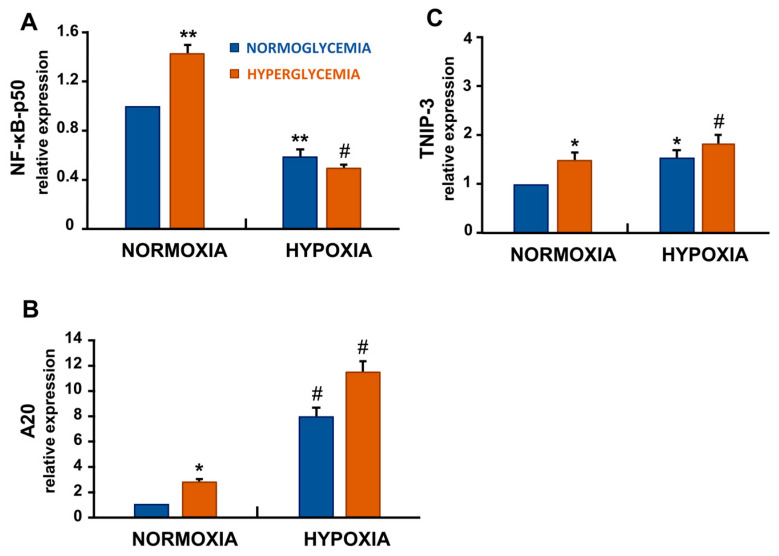
Hypoxia and combined hypoxia with high glucose downregulated NF-κB-p50 expression while upregulating A20 and TNIP-3, two negative regulators of the NF-κB pathway. High glucose increased NF-κB-p50 mRNA expression, while hypoxia, alone or in combination with high glucose, showed a marked reduction in NF-κB-p50 mRNA levels (**A**). In contrast, A20 and TNIP-3 mRNA expression (**B**,**C**) increased significantly, with the strongest induction observed with both stressors. Data represent mean ± SD of three independent experiments, each performed in duplicate [* *p* < 0.05, ** *p* < 0.01 and # *p* < 0.001 compared with control (normoxia with 5.5 mM glucose)].

To further explore the unexpected SASP response, we analysed the transcriptional profile of NF-κB-p50, a key regulator of pro-inflammatory mediators including IL-6, IL-8, and MCP-1 (Figure 4A). Hyperglycemia significantly increased NF-κB-p50 transcript levels compared with the normoglycemic control (*p* < 0.01). In contrast, NF-κB-p50 mRNA expression was markedly downregulated under hypoxia (*p* < 0.01), with the most pronounced suppression observed under combined hyperglycemic and hypoxic conditions (*p* < 0.001) (Figure 4A).

As shown in Figure 4B,C, A20 and TNIP-3, two well-established negative regulators of the NF-κB pathway, were significantly upregulated in ECs exposed to hyperglycemia compared with the normoglycemic control (*p* < 0.05 for both). Moreover, hypoxia alone increased A20 and TNIP-3 transcript levels (*p* < 0.001 and *p* < 0.05, respectively), and their expression was further elevated under combined hyperglycemic and hypoxic conditions (*p* < 0.001 for both transcripts) relative to normoxic, normoglycemic controls (Figure 4B,C).

## 4. Discussion

A progressive decline in oxygen availability is a hallmark of aging and metabolic diseases such as diabetes [11]. ECs are particularly vulnerable to the combined effects of hypoxia, chronic hyperglycemia, and oxidative stress, conditions that commonly coexist in the microenvironments of aging and diabetes. These stressors can accelerate cellular senescence, a stable state of growth arrest associated with transcriptional reprogramming, metabolic alterations, and often the secretion of pro-inflammatory factors [12].

We confirmed that ECs exposed to hypoxia and high glucose exhibited a strong senescent phenotype, evidenced by upregulation of cyclin-dependent kinase inhibitors p21 and p16, and increased β-galactosidase activity, consistent with the classical hallmarks of senescence [3].

Unexpectedly, this form of senescence was not accompanied by a canonical SASP, which is typically characterised by an NF-κB–driven pro-inflammatory secretome, including IL-6, IL-8, and MCP-1 [3,24]. Instead, the SASP appeared suppressed and was associated with a transcriptional downregulation of the NF-κB p50 subunit, indicating an attenuation of NF-κB activity. Notably, we detected a significant increase in the transcriptional expression of A20 and, for the first time under these experimental conditions, a clear induction of TNIP-3 expression.

Both A20 and TNIP-3 act as negative regulators of NF-κB by modulating signalling events upstream of this pathway [25,26,27].

Unlike A20, which possesses both deubiquitinase and E3 ligase activity, TNIP-3 is a non-enzymatic adaptor that primarily functions as a scaffold, facilitating interactions between A20 and ubiquitinated signalling intermediates [27,28]. In addition to cooperating with A20, TNIP-3 can also suppress NF-κB independently, for instance by stabilising STAT1 and attenuating pro-inflammatory signalling in specific contexts [29].

Hypoxia has been shown to activate the IKK/NF-κB pathway, promoting p65 nuclear translocation and the transcription of A20 [30]. Corresponding increases in A20 protein levels have been confirmed by Western blot in endothelial models after 6–12 h of hypoxic exposure, and pharmacological inhibition of NF-κB prevents this induction, indicating a causal link between NF-κB activation and A20 expression [25,26]. Furthermore, HIF-1α stabilisation can mimic the effect of hypoxia on A20 transcription, suggesting potential cooperation between HIF-1α and NF-κB in regulating A20 [31]. Although TNIP-3 has received less attention in this context, its established role as a negative regulator of NF-κB signalling supports the hypothesis that its expression may also be enhanced by hypoxia-induced NF-κB activation. However, the precise molecular mechanisms linking hypoxia to TNIP-3 induction remain to be fully elucidated. Further studies are needed to confirm whether these transcriptional changes translate into protein modulation and functional effects on cytokine release or endothelial senescence.

As expected, our experimental model showed an upregulation of the angiogenetic factor VEGF-A under hypoxic conditions. However, this response was accompanied by a pronounced reduction in YAP-1 expression—a key effector of the Hippo pathway crucial for sustaining EC proliferation and structural integrity.

Acute and brief hypoxia is generally reported to activate the Hippo pathway effector YAP-1 [32,33]. This activation typically occurs through post-translational mechanisms that reduce YAP-1 phosphorylation and promote its nuclear accumulation. In this active state, YAP functions as a transcriptional co-activator that enhances the expression of angiogenic genes, including VEGF itself, thereby establishing a positive feedback loop that supports endothelial proliferation and angiogenesis [34].

Another study identified YAP and TAZ as pivotal mediators of VEGF–VEGFR2 signalling [33]. EC loss of YAP/TAZ compromises vascular development and leads to embryonic lethality. VEGF activates YAP/TAZ via actin cytoskeleton remodelling, establishing a feedforward loop that sustains angiogenesis, while YAP/TAZ deficiency impairs VEGFR2 trafficking and endothelial function [33].

Nevertheless, in ECs exposed to severe hypoxic conditions, we observed a marked upregulation of VEGF accompanied by a transcriptional downregulation of YAP-1. This finding aligns with the recent study by Angom et al. [35] demonstrating that prolonged hypoxic stress in ECs induces increased expression of VEGF and its receptor VEGFR-1, while concurrently suppressing YAP-1 transcription. On the other hand, they demonstrated that YAP-1 knockdown promoted EC senescence, highlighting a critical role for YAP-1 in maintaining EC homeostasis [35,36]. Importantly, VEGFR-1 silencing modulated YAP-1 expression, revealing a previously unrecognised VEGFR-1–YAP-1 regulatory axis involved in hypoxia-induced EC senescence [35].

In high-glucose conditions, YAP/TAZ activity is also modulated in a time-dependent manner: acute hyperglycemia enhances YAP activation and VEGF expression, whereas prolonged hyperglycemic exposure or combined hypoxic and hyperglycemic conditions can attenuate YAP signalling, possibly through redox imbalance and altered cytoskeletal dynamics [24]. Therefore, the reduction in YAP-1 observed in our model, despite increased VEGF-A, may represent a compensatory mechanism aimed at preserving endothelial homeostasis under sustained hypoxic and metabolic stress.

Our findings identify a distinctive form of endothelial senescence triggered by combined hypoxic and hyperglycemic stress, which diverges from the classical proinflammatory SASP profile. This phenotype is characterised by: (1) growth arrest, as indicated by increased p21, p16, and SA-β-gal activity; (2) attenuated NF-κB–driven inflammation, mediated by the upregulation of A20 and TNIP-3; and (3) altered expression of vascular regulators, including VEGF-A upregulation and YAP-1 suppression.

This non-canonical phenotype may reflect context-dependent endothelial responses in tissues exposed to reduced perfusion or oxygen availability—such as the diabetic retina, renal glomeruli, or ischemic myocardium. Its potential impact on inflammation, microvascular structure, or cellular survival under persistent stress conditions remains to be determined and warrants further investigation.

Our data thus support the emerging concept that not all senescence is equal: the senescence response can be modulated by the cellular context and upstream stressors to yield diverse functional outcomes. In support of all this, Mongiardi et al. [37] examined how hypoxia affects ECs with different senescent states. They found that the hypoxic response is strongly influenced by the type of senescence, particularly in the modulation of the SASP. Each model showed distinct transcriptional and inflammatory profiles, indicating that hypoxia shapes angiogenic and inflammatory responses in a stimulus-specific manner [37].

This highlights the importance of re-evaluating the therapeutic targeting of senescence in vascular diseases. Inhibiting senescence per se may not be beneficial if the senescent phenotype is reparative rather than deleterious. Instead, modulation of SASP components, or the A20/TNIP-3 axis, may allow fine-tuned regulation of endothelial responses.

From a translational perspective, pharmacologic induction of A20 and TNIP-3 expression could serve as a therapeutic strategy to limit endothelial inflammation without abrogating essential stress responses. Conversely, dysregulation of these inhibitory pathways may underlie pathological endothelial senescence in chronic disease states.

We acknowledge the limitation that our current findings on TNIP-3 and A20 are based solely on mRNA expression analysis, without protein-level confirmation or assessment of downstream functional effects. Future studies evaluating temporal protein expression and employing functional approaches, such as TNIP-3 or A20 silencing combined with measurements of IL-6, IL-8, and MCP-1 release, as well as senescence biomarkers, will be essential to confirm the functional relevance of the A20/TNIP-3–NF-κB feedback mechanism under combined conditions of hypoxia and high glucose.

## 5. Conclusions

Our data reveal that ECs exposed to hypoxia, alone or combined with hyperglycemia, develop a non-canonical form of senescence characterized by the absence of classical SASP factors and the induction of A20 and TNIP-3. This unexpected signature expands current definitions of endothelial senescence and underscores the contextual plasticity of stress responses. Future studies will be essential to determine the functional consequences of this phenotype and to clarify whether such non-canonical programs contribute to endothelial behavior in hypoxic and metabolically compromised microenvironments, potentially informing new avenues for therapeutic exploration.

## Data Availability

The data that support the findings of this study are available from the corresponding author upon reasonable request.

## Abbreviations

The following abbreviations are used in this manuscript:

EC	Endothelial Cell
SASP	Senescence-Associated Secretory Phenotype
NF-κB-p50	Nuclear Factor-kappa B p50 subunit
SA-β-gal	Senescence-Associated β-galactosidase
p16	protein 16
p21	protein 21
IL-6	Interleukin-6
IL-8	Interleukin-8
MCP-1	Monocyte Chemotactic Protein-1
VEGF-A	Vascular Endothelial Growth Factor-A
YAP-1	Yes-Associated Protein-1
TAZ	Transcriptional co-Activator with PDZ-binding motif
A20	Tumor Necrosis Factor Alpha-Induced Protein 3 (TNFAIP3)
TNIP-3	TNFAIP3 Interacting Protein 3
